# Genome-Wide Association Study of H/L Traits in Chicken

**DOI:** 10.3390/ani9050260

**Published:** 2019-05-21

**Authors:** Bo Zhu, Qinghe Li, Ranran Liu, Maiqing Zheng, Jie Wen, Guiping Zhao

**Affiliations:** 1 Institute of Animal Sciences, Chinese Academy of Agricultural Sciences, Beijing 100193, China; boyzhubo@126.com (B.Z.); liqinghe@caas.cn (Q.L.); liuranran@caas.cn (R.L.); zhengmaiqing@caas.cn (M.Z.); wenjie@caas.cn (J.W.); 2 State Key Laboratory of Animal Nutrition, Beijing 100193, China; 3 School of Life Science and Engineering, Foshan University, Foshan 528000, China

**Keywords:** chicken, heterophils, lymphocyte, GWAS, SNP

## Abstract

**Simple Summary:**

With the continuous increase of intensive agriculture, the poultry industry has developed rapidly. Concurrently, diseases such as avian influenza, salmonella, and Newcastle disease have brought huge losses to the poultry industry. The traditional method of disease prevention and treatment includes vaccinations, but these have been linked to concerns associated with expense and meat safety. To solve these problems, genetic breeding methods can be used. In this paper, a genome-wide association analysis was linked to heterophil/lymphocyte ratio disease-resistance traits as a means through which disease damage can be mitigated.

**Abstract:**

Presently, the heterophil-to-lymphocyte (H/L) ratio is being studied extensively as a disease resistance trait. Through intricate mechanisms to identify and destroy pathogenic microorganisms, heterophils play a pivotal role in the immune defense systems of avian species. To reveal the genetic basis and molecular mechanisms affecting the H/L ratio, phenotypic and H/L data from 1650 white feather chicken broilers were used in performing a genome-wide association study. A self-developed, chicken-specific 55K chip was used for heterophils, lymphocytes, and H/L classification, according to individual genomic DNA profiles. We identified five significant single nucleotide polymorphisms (SNPs) when the genome-wide significance threshold was set to 5% (*p* < 2.42 × 10^−6^). A total of 15 SNPs obtained seemingly significant levels (*p* < 4.84 × 10^−5^). Gene annotation indicated that *CARD11* (Caspase recruitment domain family member 11), *BRIX1* (Biogenesis of ribosomes BRX1), and *BANP* (BTG3 associated nuclear protein) play a role in H/L-associated cell regulation and potentially constitute candidate gene regions for cellular functions dependent on H/L ratios. These results lay the foundation for revealing the genetic basis of disease resistance and future marker-assisted selection for disease resistance.

## 1. Introduction

With the continuous expansion of the poultry industry, production continues to be plagued with problems associated with disease. Although vaccination programs have dramatically reduced the incidence of many diseases and controlled the most prominent acute infections, they have not adequately addressed all infectious diseases [1]. Through systematic efforts, the combined impact of vaccination strategies, optimal nutrition, and genetic improvement, impressive increases in disease protection have been achieved. However, excessive use of vaccination and drugs in production is expensive and raises concerns for meat safety. Further improvement in the intrinsic resistance to disease, obtainable through the application of genetic principles and techniques, is desirable.

Avian heterophils, which are widely present in the peripheral blood of poultry, are equivalent to mammalian neutrophils in their defense-associated role against external pathogenic microorganisms [2]. Although avian heterophils are synonymous with animal neutrophils in their purpose, heterophils and neutrophils function in significantly differently ways [3,4]. Firstly, as heterophils do not secrete peroxidase and alkaline phosphatase, their antibacterial action is exerted through a non-oxidative deamination mechanism with selective cytokine activation [5]. Secondly, heterophils are capable of cell degranulation, oxidative burst, and phagocytosis. Pattern recognition receptors on the surface of heterophils interact with pathogens, recognize and exert phagocytosis, and secrete a large number of cytokines, such as beta defense molecules and leukotriene B4, and chemokines. This activates other immune defense pathways, thereby exerting immunity against disease [6,7,8].

Disease resistance in chicken can be improved through genetic selection for immunocompetence [9]. Generalized resistance to disease in birds is influenced by genetic and environmental factors, and involves both innate and acquired immunity; the latter being influenced by environmental factors to a greater degree. Lymphocytes are involved in acquired immunity [10]. As a simple index, the heterophil/lymphocyte (H/L) ratio in blood reflects immune system status [11]. Antibody titers [12] and circulating lymphocyte and macrophage numbers decrease, while the heterophil concentration increases in response to immunological challenges when low H/L ratios are observed [13].

Cell counts from blood smears have long been used to evaluate health parameters in animals. In most studies using leukocyte profiles, the focus was on the H/L ratio because this index reflects the dynamic between the main cell types [14]. The H/L ratio was initially suggested to be an indicator of stress [2], since this ratio is seen to increase when chickens are stressed. The increase in the H/L ratio has been shown to be more striking in response to the first, rather than a second, imposed stress [2]. The H/L ratio has also been used as a selection criterion for response to the Newcastle disease vaccine and general resistance to heat stress [15]. In addition, since the H/L ratio changes with different environments [16] and is associated with baseline corticosterone levels in adult birds, it is recognized as an indicator of animal welfare [17]. Consequently, following stress on the body, the change in the H/L ratio can be used as a stress index, since it is associated with body strength and stress resistance [18]. The current study is based on white feather broiler chickens farmed at the Foshan Gaoming District Xinguang Agriculture and Animal Husbandry Co., Ltd. (Foshan, China). General disease resistance mechanisms associated with the H/L ratio are poorly understood. Therefore, in order to lay the foundation for further analysis into the molecular mechanisms of the H/L ratio and subsequent molecular marker-assisted selection of breeding pairs, genomic DNA typing was achieved using a self-developed 55K single nucleotide polymorphism (SNP) chip (Beijing Compass Biotechnology Co., Ltd., Beijing, China) and a genome-wide association study (GWAS) performed in white feather broiler chicken. In performing the GWAS, monocyte, lymphocyte, heterophil, and H/L ratio data were used to identify quantitative trait loci or functional genes that affect each cell type.

## 2. Materials and Method

### 2.1. Experimental Animals

The work was approved by the Animal Management Committee of the Institute of Animal Sciences, Chinese Academy of Agricultural Sciences (IAS-CAAS, Beijing, China). Ethical approval on animal survival was given by the animal ethics committee of IAS-CAAS (approval number: IASCAAS-AE20140615).

The experimental cluster is represented by the white feather broiler B line ancestral chicken of the Guangdong Foshan Xinguang Agriculture and Animal Husbandry Co., Ltd. Raised at the same facility, the resource groups were used for breeding purposes.

The chickens were reared in a fully enclosed, shaded chicken house. The house temperature was 34 to 35 °C the first day. The difference between day and night temperatures did not exceed 1 °C. The temperature was decreased by 1 °C every three days and 2 °C per week until room temperature was achieved. The chickens were exposed to 24 hours of light for three days, 23 hours of light on the fourth day, and a 1 hour decrease in light every two days until natural lighting was achieved. Humidity in the chicken house was maintained at 70–80%. Floor space available was 1 m^2^ per 3 chickens. Using corn–soybean-type diets, the nutritional levels of each generation remained unchanged. Blood was collected from the wing vein at 42 days of age with anticoagulation, achieved using anticoagulant citrate dextrose 1.32% (M/V), sodium citrate (M/V), 0.48% (M/V) citric acid, and 1.47% (M/V) glucose. Anticoagulated blood samples were stored at −20 °C for genomic DNA extraction. H/L traits were individually measured at 42 days of age.

### 2.2. Phenotypic Measurement

At 42 days of age, two glass slides were each smeared on a single side with 10 μL of blood, freshly obtained from the lower part of the chicken’s wing. Slides were air-dried and dyed with May–Grunwald–Giemsa stain. One hundred leukocytes, including granular (heterophils) and nongranular (lymphocytes and monocytes) components, were counted on one slide for each bird and the heterophil-to-lymphocyte ratio calculated [19]. All statistics were performed using SAS 9.2 (SAS Institute, Inc., Carly, NC, USA). Data that did not have a normal distribution underwent box–cox transformation [20].

### 2.3. Genotyping and Quality Control

Blood samples were obtained using standard venipuncture techniques. Genomic DNA was extracted from blood samples, using a standard phenol/chloroform method and genotyped with a 55K Affymetrix Axiom Chicken Genotyping Array (Affymetrix, Inc. Santa Clara, CA, USA). Genotype quality control was performed with PLINK 1.9 [21]. Samples and SNPs with call rates lower than 90% were excluded. SNP data quality indicators included the exclusion of individuals with a genotype deletion greater than 10% and SNPs with a minimum allele frequency of less than 1%. SNPs were also excluded when the deletion rates in the case and control groups were significantly different (*p* < 10%). High quality, raw genotypic data are critical to the success of a GWAS analysis. The effectiveness of the research is greatly reduced if even typing errors are as low as 1%.

### 2.4. Genome-Wide Association Analysis

Because false associations may be due to the presence of cryptographic correlations or hidden population stratification, a simple method was used to correct the number of multiple tests needed to determine the threshold for the whole genome significant/implicit association. Prior to the GWAS, principle component analysis (PCA) was performed in PLINK 1.07 [22]. Using this approach, we obtained 20,668 recommended independent tests. The genome-wide and implied *p* values were 2.42 × 10^−6^ and 4.84 × 10^−4^, respectively.

We initially performed a univariate GWAS by applying a linear mixed model to account for associations between H/L and effective SNPs, using GEMMA [23]. The statistical model applied in this study is as follows:

y = Wα + xβ + u + ε

In this expression, y denotes the phenotypic values of n samples, while W refers to a covariance matrix used to control population structure, α denotes a vector of corresponding effects that comprise the intercept, x denotes the marker genotypes, β refers to the effects of the corresponding markers, u is a vector of random polygenic effects, and ε is a vector of random residuals.

### 2.5. Gene Identification and Annotation

Annotated genes and associated SNPs whose *p* values were found to be significant by GWAS analysis following correction were identified as candidate genes [24]. BioMart was used to detect genes in specific genomic regions [25]. This software has the Gallus genome version, which is supported by the Ensemble box NCBI [26].

## 3. Results

### 3.1. Phenotypic Description and Genetic Parameters

Means and standard deviations for H/L, monocytes, heterophils, and lymphocytes are presented in Table 1. Data that did not have a normal distribution underwent box–cox transformation.

### 3.2. Population Structure

**Figure 1:**
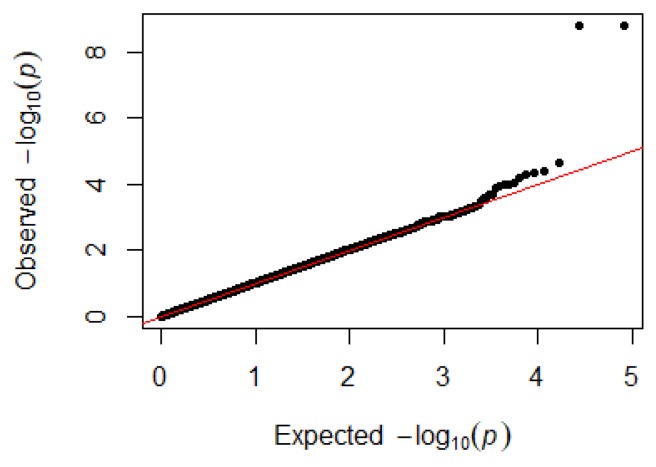
Quantile–quantile plots of *p*-values for H/L. The x-axis is the expected −log10 *p*-value and the y-axis is the observed −log10 *p*-value.

**Figure 2:**
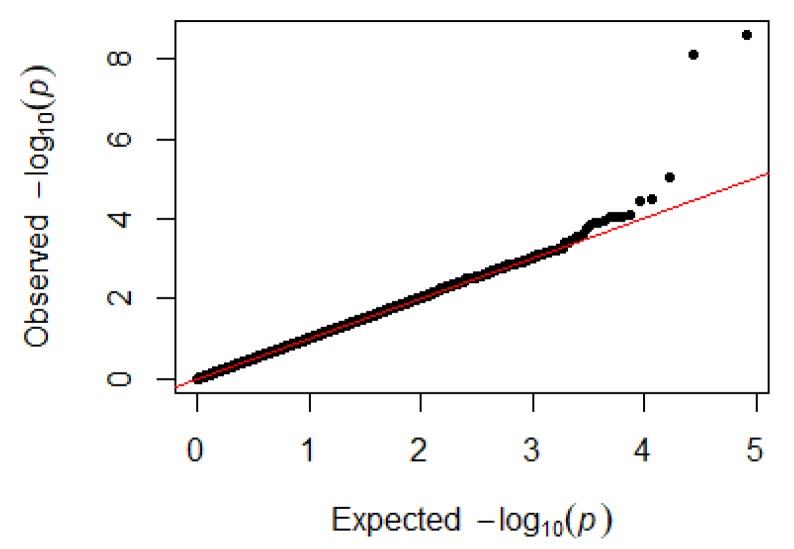
Quantile–quantile plots of *p*-values for heterophils. The x-axis is the expected −log10 *p*-value and the y-axis is the observed −log10 *p*-value.

**Figure 3:**
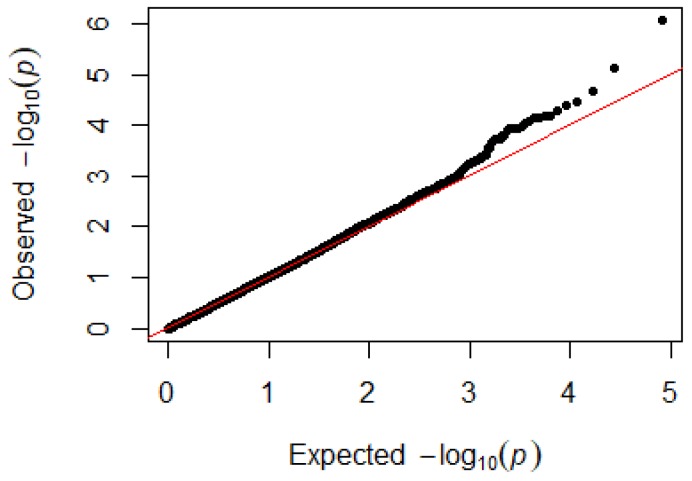
Quantile–quantile plots of *p*-values for lymphocytes. The x-axis is the expected −log10 *p*-value and the y-axis is the observed −log10 *p*-value.

**Figure 4:**
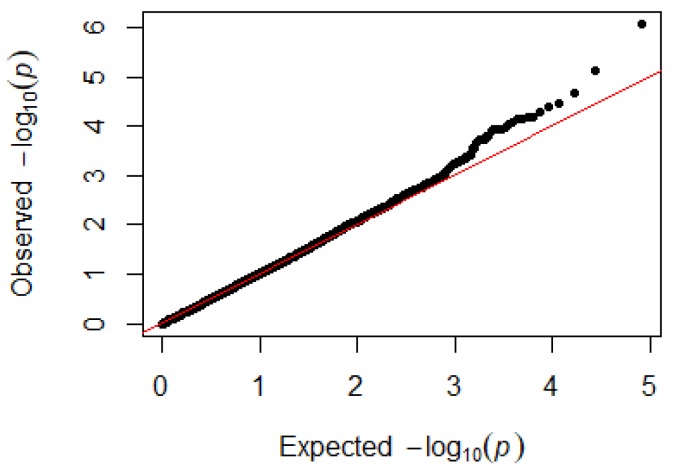
Quantile–quantile plots of *p*-values for monocytes. The x-axis is the expected −log10 *p*-value and the y-axis is the observed −log10 *p*-value.

Since GEMMA (v. 0.98, University of Michigan, Ann Arbor, Michigan, USA) is based on the hybrid model and cannot avoid the group stratification problem, it is necessary to conduct stratified tests on the test population. Q–Q plots performed on the three traits indicated that the χ^2^ distribution calculated by SNP correlation analysis did not deviate from the null hypothesis test distribution. The χ^2^ value of the significant SNP locus observations is above the expected χ^2^ value. This showed that there was no group stratification in the population under study and that the correlation analysis results of this analytical method were reliable (Figure 1, Figure 2, Figure 3 and Figure 4).

### 3.3. H/L Ratio

**Figure 5:**
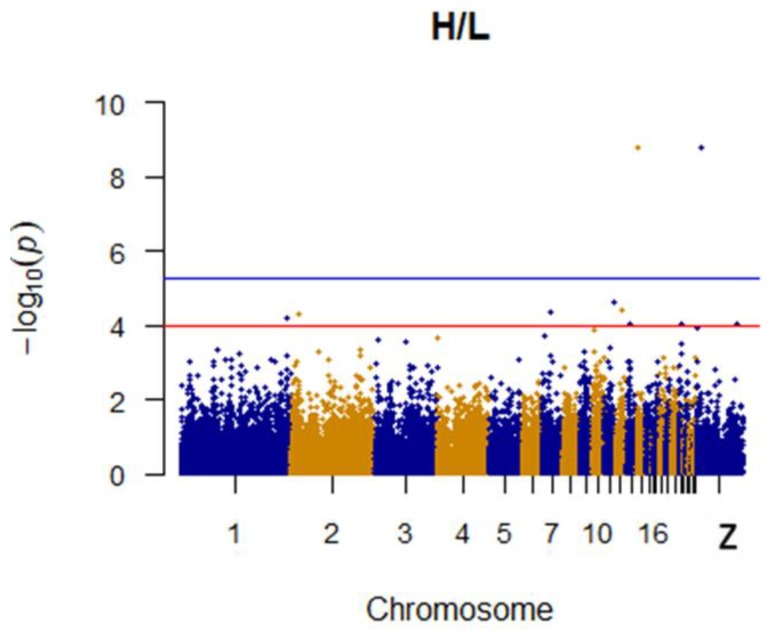
Manhattan plot for H/L in chicken. The x-axis is the position of each single nucleotide polymorphism (SNP) on the chicken chromosomes 1–28 and linkage group and the y-axis is the −log10 *p*-value.

It was found that two SNPs were significantly associated with the H/L ratio. The most significant of these is located on chromosome 14 and associated with *CARD11* (Caspase recruitment domain family member 11). A second SNP is presumably associated with the H/L ratio. Specific details concerning SNPs identified as being linked to the H/L ratio and their associated genes are shown in Table 2. The Manhattan plot for the H/L ratio is shown in Figure 5.

### 3.4. Heterophils and LYMPHOCYTES

**Figure 6:**
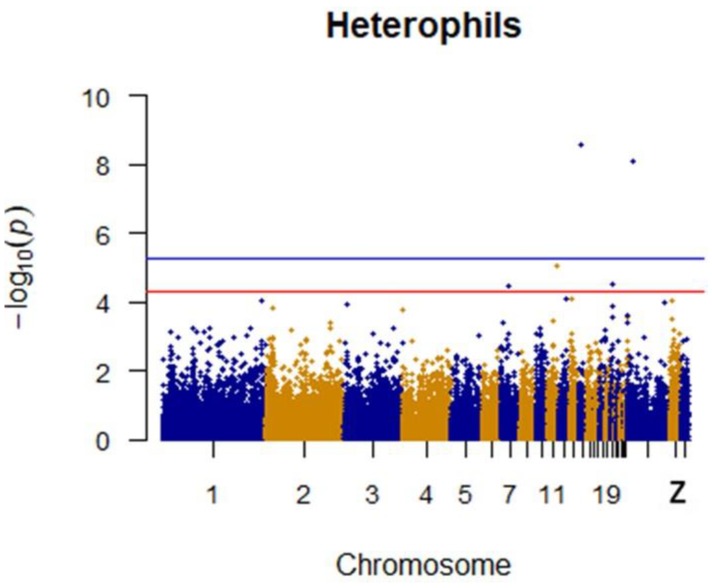
Manhattan plot for heterophils in chicken. The x-axis is the position of each single nucleotide polymorphism (SNP) on the chicken chromosomes 1–28 and linkage group and the y-axis is the −log10 *p*-value.

**Figure 7:**
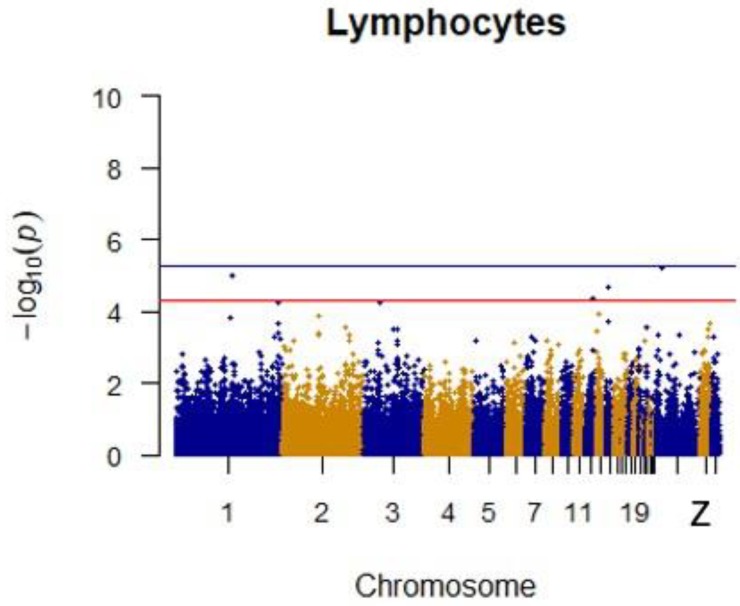
Manhattan plot for lymphocytes in chicken. The x-axis is the position of each single nucleotide polymorphism (SNP) on the chicken chromosomes 1–28 and linkage group and the y-axis is the −log10 *p*-value.

After quality control, a total of 1500 42-day-old broiler chickens were analyzed. Manhattan mapping revealed one SNP on chromosome 2 and one SNP on chromosome 3 that were significantly associated with heterophils. A further three SNPs were located on chromosomes 14, Z, and 11. Two heterophil-associated genes were found. Located on chromosome 1 and the Z chromosome, respectively, two novel SNPs were significantly associated with lymphocytes. Two SNPs located on chromosomes 14 and 21 appeared to be associated with lymphocytes. Specific details are shown in Table 3. Manhattan plots for heterophils and lymphocytes are shown in Figure 6 and Figure 7, respectively.

### 3.5. Monocytes

**Figure 8:**
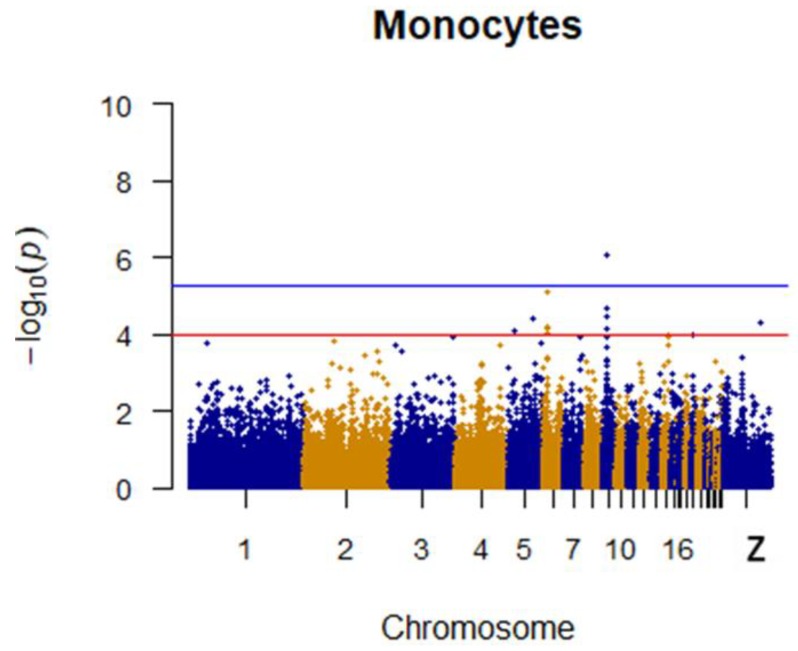
Manhattan plot for monocytes in chicken. The x-axis is the position of each single nucleotide polymorphism (SNP) on the chicken chromosomes 1–28 and linkage group and the y-axis is the −log10 *p*-value.

Two SNPs significantly associated with monocytes and two SNPs with suggestive associations to monocytes were located on chromosomes 9, 6, and 5. SNPs on chromosome 6 were linked to multiple loci, which included the *TMEM26*(Transmembrane protein 26) and *RHOBTB1*(Rho related BTB domain containing 1). Specific details are shown in Table 4. The Manhattan plot for monocytes is shown in Figure 8.

## 4. Discussion

The H/L ratio in chicken peripheral blood has been widely accepted as a reliable and accurate physiological indicator of chicken stress response [15]. With high or low temperature, excessive NH_3_ exposure, bacterial infection, and other stress reactions, the number of lymphocytes decreases while the number of heterophils increases [27,28]. The number and proportion of heterophils and lymphocytes are highly heritable, with a heritability estimated to exceed 0.5 [29], indicating that these traits should respond well to selection. In this study, one fairly correlated SNP and two significantly associated SNPs were linked to the H/L ratio. The SNP with the most significant association to the H/L ratio is located 103.4 kb downstream of the *CARD11* gene on chromosome 14. The protein encoded by this gene belongs to the membrane-associated guanylate kinase family, a class of proteins that are used as molecular scaffolds. Polyprotein complexes are assembled in specific regions of the plasma membrane. This protein is likewise a member of the CARD protein family, as defined by carrying a characteristic caspase-associated recruitment domain (CARD). This protein has a domain structure similar to the CARD14 protein. The CARD domain of both proteins has been shown to specifically interact with BCL10 [30], a protein that has been recognized for acting as a positive regulator of apoptosis and *NF-κB* (Nuclear factor kappa B subunit 1) activation [31,32]. When expressed in cells, BCL10 activates NF-κB and induces phosphorylation of *BCL10* [30,33,34]. Siwekidentified *CARD11* as a candidate gene for the quantitative trait locus linked to the immune response in chicken [35]. Slawinska echoed this finding [36].

Another significant site located 0.3 kb downstream of the *BRIX1 (Biogenesis of ribosomes BRX1)* gene on the Z chromosome was found. *BRIX1* (ribosomal biogenesis protein BRX1) is a protein-coding gene associated with the gastric cancer network pathway and neural development of the chicken brain [37]. 

There is also a suggestion that a heterophil-associated SNP is located in an intronic region of the *BANP* gene on chromosome 11. This gene encodes a protein that binds to the matrix attachment region to form a complex with p53. So doing, it negatively regulates p53 transcription and acts as a tumor suppressor and cell cycle regulator. Binding to the scaffold/matrix attachment region β occurs in an ATC-rich DNA sequence, located upstream of the T cell receptor β enhancer region. V(D)J recombination during T cell development is controlled by inhibition of the T cell receptor β enhancer function. By recruiting *HDAC*1(Histone deacetylase 1) to its promoter region, H3K9ac, H3S10ph, and H4K8ac levels are reduced to inhibit cyclin D1 transcription. This promotes phosphorylation and nuclear accumulation of TP53 Ser-15, leading to cell cycle arrest by similarity [38].

In addition, an SNP that was significantly associated with lymphocytes is located 252.4 kb upstream of C1H21ORF91 on chromosome 1 and is involved in staphylococcal toxemia [39].

The nearest gene to one of the SNPs that is significantly associated with monocytes is EPHA4. This gene belongs to the heparin receptor subfamily of the protein–tyrosine kinase family. EPH receptor-associated molecules are involved in mediating developmental events, particularly in the nervous system. Diseases associated with *EPHA4* (Ephrin type-A receptor 4) include lung mucoepidermoid carcinoma and Duane retraction syndrome [40].

*TMEM2*6 (Transmembrane protein 26), which encodes a protein containing multiple transmembrane helices, was also described as being linked to the SNP significantly associated with monocytes. It is a selective surface protein marker for beige fat cells that can coexist with classical brown fat cells in brown adipose tissue [41].

A third monocyte-associated SNP was located 10.0 kb upstream of *RASA2* (RAS P21 protein activator 2). The protein encoded by this gene is a member of the general amino-acid permease 1 family of GTP1 (Guanosine triphosphate1) activating proteins. This gene product stimulates GTPase (Guanosine triphosphate enzyme) activity in normal RAS p21 molecules, but does not stimulate its carcinogenic counterpart. As an inhibitor of RAS function, this protein enhances the weak intrinsic GTPase activity of the RAS protein, resulting in an inactive GDP binding form of RAS, which controls cell proliferation and differentiation [42].

On chromosome 6, *TMEM26* and *RHOBTB1* were identified as genes linked to the monocyte-associated SNPs. While *TMEM26* encodes proteins containing multiple transmembrane helices, which act as selective surface protein markers for brown/beige fat cells that can coexist with classical brown fat cells in brown adipose tissue [41,43,44], the protein encoded by *RHOBTB1* belongs to the Rho family of the small GTPase superfamily. It contains a GTPase domain, a proline-rich region, a tandem of two BTB (wide complex, tram, and bric-a-brac) domains, and a conserved C-terminal region. This protein plays a role in small GTPase-mediated signal transduction and organization of the actin filament system [38]. In this region, these two genes appear frequently, and are reportedly associated with genes linked to feed conversion rate and eggshell weight [45].

The final SNP site under discussion is located 18.9 kb downstream of the *PID*1 (Phosphotyrosine interaction domain containing 1) gene and is associated with an increased proliferation of pre-adipocytes without affecting adipocyte differentiation [46].

## 5. Conclusions

In conclusion, considering their physical location and biological function, the four novel genes identified in this study appear to be promising candidate genes for H/L-associated traits. Chromosome 6 may also be an important candidate region for monocytes. Due to its connection to desirable immune traits, results from this study may be useful for subsequent studies to reveal the mechanism of action associated with the H/L ratio. Since the measurement of the H/L ratio from blood smears is simple and inexpensive, individuals with low H/L ratios can be readily identified for selection and, along with other desirable traits, contribute to improved disease resistance. Future experiments should include replicating the candidate genes identified in this study to provide transcriptomic data from artificial infection experiments.

## Figures and Tables

**Table 1:** Descriptive statistics of phenotypic data.

Traits	Mean	SD	Deviation	Min	Max	CV1 (%)
Monocytes (M, n)	3	3	7	0	17	87
Heterophils (H, n)	27	9	88	2	64	34
Lymphocytes (L, n)	73	18	333	21	88	25
H /L (H/L, %)	40	20	0	0	1.7	50

Abbreviations: Mean = arithmetic mean; SD = standard deviation; Min = minimum; Max = maximum; CV = coefficient of variation.

**Table 2:** Single nucleotide polymorphisms (SNPs) with genome-wide significance for H/L traits.

Traits	Chromosome	SNP ID	Position ^1^ (BP)	*p*_Wald	Nearest Gene	Distance ^2^
H/L	14	rs15005639	3332117	1.55 × 10^−^^9^	*CARD11*	D103.6
H/L	Z	rs314642216	10570600	1.59 × 10^−9^	*BRIX1*	D3.4
H/L	11	rs14028611	18280341	2.27 × 10^−5^	*BANP*	within
H/L	12	New	12911436	4.16 × 10^−5^	*PTPRG*	within
H/L	7	rs312628231	15894092	4.32 × 10^−5^	*NFE2L2*	D10.6

^1^ SNP positions are obtained from ENSEMBLE. ^2^ U = upstream, D = downstream. The unit of the distance is kb. *CARD11* = Caspase recruitment domain family member 11). *BRIX1* = Biogenesis of ribosomes. *BANP* = BTG3 associated nuclear protein. *PTPRG* = Protein tyrosine phosphatase receptor type G. *NFE2L2* = Nuclear factor, erythroid 2 like 2.

**Table 3:** SNPs with genome-wide significance for heterophil and lymphocyte traits.

Traits	Chromosome	SNP ID	Position ^1^ (BP)	*p*_Wald	Nearest Gene	Distance ^2^
Heterophils	14	rs15005639	3332117	2.54 × 10^−9^	*CARD11*	D103.6
Heterophils	Z	rs314642216	10570600	8.06 × 10^−9^	*BRIX1*	D3.4
Heterophils	11	rs14028611	18280341	8.81 × 10^−6^	*BANP*	within
Heterophils	3	rs316444238	3269267	3.13 × 10^−5^	*PIK3CD*	U192.3
Heterophils	2	rs312628231	15894092	3.52 × 10^−5^	*NFE2L2*	D10.6
Lymphocytes	Z	rs314642216	10570600	5.88 × 10^−6^	*BRIX1*	U3.4
Lymphocytes	1	New	1.02E+08	9.87 × 10^−6^	*C1H21ORF91*	U2524.9
Lymphocytes	14	rs15005639	3332117	2.14 × 10^−5^	*CARD11*	D103.6
Lymphocytes	21	New	12911436	4.30 × 10^−5^	*PTPRG*	within

^1^ SNP positions are obtained from ENSEMBLE. ^2^ U = upstream, D = downstream. The unit of the distance is kb. *CARD11* = Caspase recruitment domain family member 11. *BRIX1* = Biogenesis of ribosomes BRX1. *BANP* = BTG3 associated nuclear protein. *PIK3CD* = Phosphatidylinositol-4,5-bisphosphate 3-kinase catalytic subunit delta. *NFE2L2* = Nuclear factor, erythroid 2 like 2. *BRIX1* = Biogenesis of ribosomes BRX1. *C1H21ORF91* = Chromosome 21 open reading frame 91. *CARD11* = Caspase recruitment domain family member 11. *PTPRG* = Protein tyrosine phosphatase receptor type G.

**Table 4:** SNPs with genome-wide significance for monocyte traits.

Traits	Chromosome	SNP ID	Position ^1^ (BP)	*p*_wald	Nearest Gene	Distance ^2^
Monocytes	9	New	7719173	8.62 × 10^−7^	*EPHA4*	within
Monocytes	6	rs313943680	8556872	7.59 × 10^−6^	*TMEM26* *RHOBTB1*	within U87.7
Monocytes	9	New	10113376	2.08 × 10^−5^	*RASA2*	U100.5
					*PID1*	U189.3
Monocytes	9	New	8301605	3.42 × 10^−5^	*WDFY1*	within
Monocytes	5	rs313016555	44638604	4.01 × 10^−5^	*CPSF2*	within

^1^ SNP positions are obtained from ENSEMBLE. ^2^ U = upstream, D = downstream. The unit of the distance is kb. *EPHA4* = Ephrin type-A receptor 4. *TMEM26* = Transmembrane protein 26. *RHOBTB1* = Rho related BTB domain containing 1. *RASA2 =* RAS P21 protein activator 2. *PID1* = Phosphotyrosine interaction domain containing 1. *WDFY1* = WD repeat and FYVE domain containing 1. *CPSF2* = Cleavage and polyadenylation specific factor 2.
